# Diagnostic Process in Rare Diseases: Determinants Associated with Diagnostic Delay

**DOI:** 10.3390/ijerph19116456

**Published:** 2022-05-26

**Authors:** Juan Benito-Lozano, Greta Arias-Merino, Mario Gómez-Martínez, Alba Ancochea-Díaz, Aitor Aparicio-García, Manuel Posada de la Paz, Verónica Alonso-Ferreira

**Affiliations:** 1 Institute of Rare Diseases Research (IIER), Instituto de Salud Carlos III, 28029 Madrid, Spain; jbenito@isciii.es (J.B.-L.); garias@isciii.es (G.A.-M.); mario.gomez@isciii.es (M.G.-M.); mposada@isciii.es (M.P.d.l.P.); 2 Universidad Nacional de Educación a Distancia (UNED), 28015 Madrid, Spain; 3 Spanish Federation of Rare Diseases (FEDER), 28009 Madrid, Spain; direccion@enfermedades-raras.org; 4 The State Reference Center for Assistance to People Living with Rare Diseases and Their Families (CREER), Centro de Referencia Estatal de Atención a Personas con Enfermedades Raras y sus Familias, Dependiente del IMSERSO, 09001 Burgos, Spain; aapariciog@imserso.es; 5 Centro de Investigación Biomédica en Red de Enfermedades Raras (CIBERER), 28029 Madrid, Spain

**Keywords:** rare diseases, diagnostic odyssey, diagnostic delay, time to diagnosis, diagnostic process, Spain, public health

## Abstract

Many people living with rare disease (RD) report a difficult diagnostic process from the symptom onset until they obtain the definitive diagnosis. The aim of this study was thus to ascertain the diagnostic process in RDs, and explore the determinants related with having to wait for more than one year in this process (defined as “diagnostic delay”). We conducted a case–control study, using a purpose-designed form from the Spanish Rare Diseases Patient Registry for data-collection purposes. A descriptive analysis was performed and multivariate backward logistic regression models fitted. Based on data on 1216 patients living with RDs, we identified a series of determinants associated with experiencing diagnostic delay. These included: having to travel to see a specialist other than that usually consulted in the patient’s home province (OR 2.1; 95%CI 1.6–2.9); visiting more than 10 specialists (OR 2.6; 95%CI 1.7–4.0); being diagnosed in a region other than that of the patient’s residence at the date of symptom onset (OR 2.3; 95%CI 1.5–3.6); suffering from a RD of the nervous system (OR 1.4; 95%CI 1.0–1.8). In terms of time taken to see a specialist, waiting more than 6 months to be referred from the first medical visit was the period of time which most contributed to diagnostic delay (PAR 30.2%). In conclusion, this is the first paper to use a collaborative study based on a nationwide registry to address the diagnostic process of patients living with RDs. While the evidence shows that the diagnostic process experienced by these persons is complex, more studies are needed to determine the implications that this has for their lives and those of their families at a social, educational, occupational, psychological, and financial level.

## 1. Introduction

Rare diseases (RDs) are those whose prevalence is below 5 cases per 10,000 inhabitants in the European Community and Orphanet has described more than 6000. Diagnostic delay in RDs is a problem which has an impact on the lives, not only of the affected persons and their families, but also of society as a whole. From the time of symptom onset until a definitive diagnosis is received, the RD diagnostic process may involve visits to different specialists, numerous tests (sometimes unnecessary), travel to health centres other than the patient’s usual clinic, moving house, inappropriate treatments, hospitalisations, or the performance of surgical interventions, among other problems [1]. Furthermore, this does not lead to a correct diagnosis in every case, with the ensuing risk of potential iatrogenic effects. These determinants form part of a process which gives rise to increased stress, anxiety, and uncertainty about the patient’s future—something experienced by the whole family—in what has become known as “a diagnostic odyssey” [2,3]. Moreover, there is a lack of sufficiently detailed information about this diagnostic process and its determinants for RDs as a whole, which would allow for the necessary quantification and ensuing implementation of preventive action targeted at resolving the problem, partly or totally. While there are studies, essentially survey-based, which are not supported by related clinical data that would make it possible to validate the data collected, others focus on specific RDs and make no attempt to perform an in-depth analysis of the complete diagnostic process [4,5,6,7,8,9]. 

Based on the Aarhus Statement, which lays down the key time points and periods of the diagnostic process, the following three time periods can be distinguished [10]: (i) the period running from symptom onset until first medical visit, generally in a primary health care setting; (ii) the period that elapses between first medical visit and referral to specialised care; (iii) the period covering the time from referral to a specialist until reaching a definitive diagnosis, which could be further subdivided into, firstly, the time from referral until being seen by the specialist, and secondly, the time from that first specialist appointment until obtaining the diagnosis. 

Among its goals for 2027, the International Rare Diseases Research Consortium (IRDiRC) requires that all known RDs be diagnosed within a maximum of one year from the date on which medical advice on the symptoms is first sought [11]. As there is no standardised definition of diagnostic delay, this Consortium’s guideline can be adopted as a criterion, according to which a wait of more than one year between these two key time points could be regarded as a delay in diagnosis. Similarly, although there is no clear list of causes that contribute to such a diagnostic delay, the most cited of these include: (i) the lack of scientific knowledge surrounding RDs as a whole, which can be frustrating; (ii) the presence of widely varying non-specific symptoms that overlap between different clinical entities; (iii) the time spent in attending the different medical appointments, undergoing tests and obtaining results; (iv) the unavailability of ad hoc diagnostic tests [12]. For their part, health systems add intrinsic difficulties arising from the way they are organised and operate, and their procedures for authorising the transfer of patients between centres, all of which have an impact on this diagnostic delay. Further influential factors are a lack of sufficient staff, difficulties in finding specialised centres, and distances between them, due to them being generally situated in other regions [13]. 

Accordingly, the aim of this study was to ascertain the diagnostic process to which people living with RDs in Spain are subjected, and to explore the determinants linked to the delay in obtaining a diagnosis of an RD.

## 2. Materials and Methods

### 2.1. Study Design

We designed a case–control study using the following operational case definition: any patient whose diagnosis took more than one year from the date of his/her first medical visit, due to symptoms attributable to his/her RD [11]. Controls were defined as patients who managed to obtain a diagnosis of their disease within one year from their first medical visit.

As our sole data-source and inclusion criterion, we used patients who were entered on the Spanish Rare Diseases Patient Registry at the Carlos III Health Institute (Instituto de Salud Carlos III), as of 1 January 2022, and who voluntarily agreed to participate in the study, provided in every case that they were resident in Spain [14]. Study participation was not limited by age, sex, or type of RD (Table S1). In those cases where patients’ age or capacity rendered it impossible for them to answer, a guardian, relative, or caregiver was allowed to answer on their behalf. We excluded patients whose diagnostic process had taken place in another country.

### 2.2. Form Data Collection

The study data were sourced from a Spanish Rare Diseases Patient Registry form which was purpose-designed for this project and specifically geared to describing the diagnostic process [15]. The following participating institutions actively helped to promote patient registration at the Registry and disseminate the study: the Spanish Rare Disease Federation (Federación Española de Enfermedades Raras/FEDER); the State Reference Centre for the Care of People with Rare Diseases and their families (Centro de Referencia Estatal de Enfermedades Raras/CREER), which comes under the IMSERSO; the Institute of Rare Disease Research.

Computer-assisted web interviewing methodology [16] was completed with questionnaires adapted for visually handicapped persons. To prevent participation biases due to the digital divide and the COVID-19 pandemic, telephone surveys were administered to whoever requested them, as well as face-to-face surveys. 

### 2.3. Measures

For analysis purposes the following variables were included, relating to: (i) the patient: sex, place of residence, type of RD (classified by organ or main system affected as per ICD10 criterion), and the dates (month/year) of symptom onset, first medical visit, and obtaining the definitive diagnosis. The last two dates were used to calculate the variable ‘diagnostic delay’, but where these dates could not be obtained, the qualitative question referring to this same time period was used instead; (ii) symptom onset of patient’s disease: age, decade, type of first medical visit, time elapsed between symptom onset and first medical visit, time elapsed between the latter and referral to the specialist, and time elapsed between referral and attending the first appointment; (iii) process until diagnosis: travel (including destination and number of journeys), changes of address, diagnosis in the same place as symptom onset or first hospital attended, specialists visited and tests performed (including frequency and number of different specialists/tests), hospitalisations and surgical interventions. Data were collected for the period before and immediately after diagnosis, up until one year after diagnosis had been obtained; (iv) diagnosis: time to diagnosis from date of first medical visit, age, place, and definitive or confirmatory test.

### 2.4. Statistical Analysis

We performed a descriptive analysis including the frequencies of presentation of the variables involved, comparing cases and controls. Univariate logistic regression and a multivariate model were used to calculate the risks of each variable. The dependent variable of the main model was obtaining diagnosis in under (=0) or over a year (=1), and taking under (=0) or over 6 months (=1) from symptom onset to the first medical visit was likewise modelled. The categorical variables were pre-tested as dummy variables. The multivariate model was adjusted by means of a backward process, with the criterion for the elimination of variables being the fact that the 95% confidence intervals (95%CI) of the odds ratios (ORs) of each variable did not include unity and that the difference between the likelihood ratios of the models was greater or not greater than −2logLR. The dummy variables were included in the initial multivariate model if some of their strata in the univariate model showed ORs with 95%CI not containing unity. In both cases, the resulting variables are shown in the final model, with a footnote indicating the variables for which they were adjusted. Two of the authors separately replicated the analyses using the SPSS v27 and Stata 14 software programmes, in order to confirm that the results matched. Lastly, we calculated the Population Attributable Risk (PAR) of taking more than 6 months in each of the 3 time periods, i.e., from symptom onset to first medical visit, from first medical visit to referral to a specialist, and from referral to having the appointment, comparing cases and controls and type of RD [17].

## 3. Results

Data were obtained on a total of 1232 persons who participated in the Spanish Rare Diseases Patient Registry’s study on diagnostic delay. Application of the exclusion criterion and data-screening yielded complete and consistent information on 1216 people living with an RD (699 cases and 517 controls; 58.8% women). 

Table 1 shows the distribution of the variables between cases and controls. The first medical visit due to symptom onset and information about the disease took place in a primary health care setting (either family doctor or paediatrician): 58.2% of cases vs. 41.0% of controls. During the search for diagnosis, 67.7% of cases and 43.5% of controls had to travel to a hospital or specialist other than those in their place of residence. Persons with a diagnostic delay had to travel to a greater extent and more often, whether within their home province, to another province in their home Autonomous Region (AR), to another AR, or to another country. Indeed, twice as many patients with a diagnostic delay were forced to travel to another AR (26.6 vs. 13.2% of controls), mainly to Madrid and Catalonia. Furthermore, RD-related hospitalisations and surgical interventions were also more frequent among persons who experienced a delay in diagnosis (34.8% were hospitalised due to their RD vs. 25.1% of controls). The frequency of visits to specialists and tests performed before obtaining diagnosis was likewise higher among those who experienced diagnostic delay. With regard to the confirmatory test, 38.6% of cases and 21.8% of controls were diagnosed through genetic testing. Lastly, once diagnosis had been obtained, most of the patients with diagnostic delay who were being treated underwent a change in treatment due to the indication furnished by the diagnosis made (59.3%), with this figure being lower among controls (38.0%).

**Figure 1:**
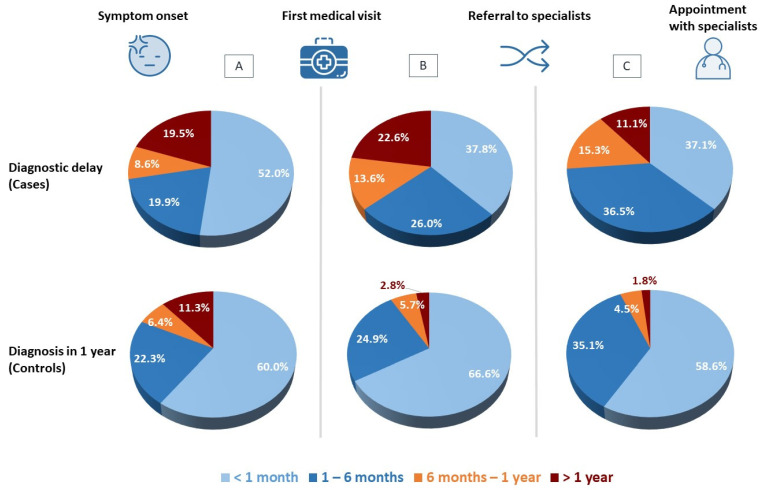
Time periods elapsed: (**A**) from symptom onset to first medical visit; (**B**) from first medical visit to referral to specialist; (**C**) from referral to appointment with specialist.

**Figure 2:**
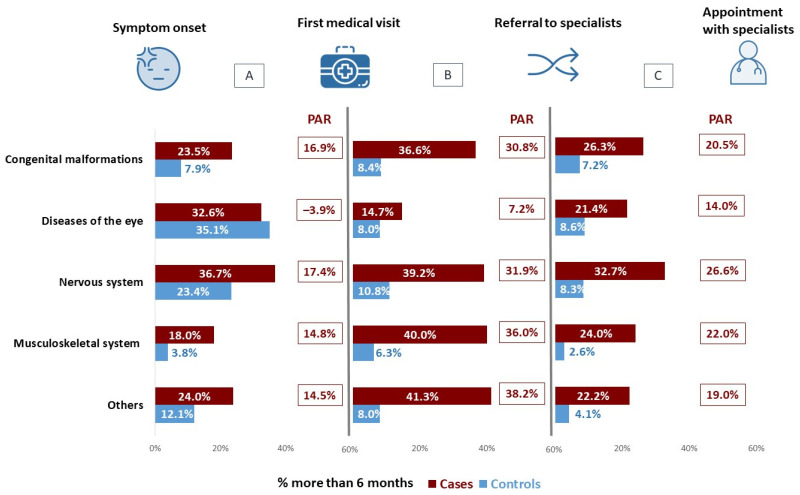
Percentage of RD-affected persons who took more than 6 months: (**A**) from symptom onset to first medical visit; (**B**) from first medical visit to referral to specialist; (**C**) from referral to appointment with specialist. PAR: Population Attributable Risk.

**Figure 3:**
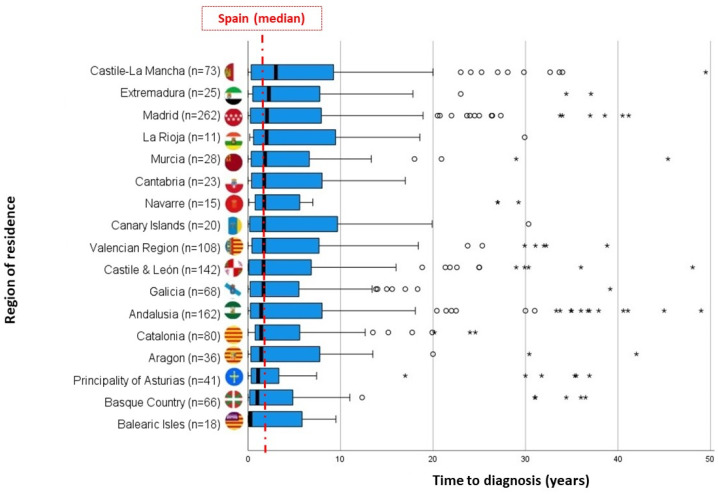
Time to diagnosis (years) by AR of RD symptom onset. Box-plot. The whisker plot at left shows (Xmin, Q1). The first part of the box (Q1, Q2). The second part of the box (Q2, Q3). The line inside the box represents the median of each AR, and the vertical red line, the median for Spain. The whisker plot at right is given by (Q3, Xmax). Circles: outliers that exceed the length of the IQR box by 1.5 units. Asterisks: outliers that exceed the length of the IQR box by 3 units.

On comparing the times elapsed between RD symptom onset, attending a first medical visit, being referred to the specialist, and having the first appointment with that specialist, persons who experienced diagnostic delay were observed to take longer in every case (Figure 1). This pattern was maintained on analysing by type of RD, especially among persons affected by diseases of the nervous system (Figure 2). For RDs as a whole, the length of time between first medical visit and referral to a specialist is the period that displays the highest attributable risk (PAR 30.2%), followed by the time between referral and appointment with the specialist (PAR 21.4%) and the time taken by patients to first seek medical advice about their symptoms (PAR 12.6%). With regard to the time taken from first medical visit to diagnosis, the great dispersion of data meant that it was impossible to observe differences by AR of residence (*p* = 0.745; Figure 3). 

According to the multivariate model shown in Table 2, the risk of taking more than 6 months to seek medical advice about symptoms was 8 times higher among persons who had initial manifestations of their RD before 1980 (OR 8.2; 95%CI 4.9–13.7), and more than twice as high among those with symptom onset in the 3 decades from 1980 to 2009. In terms of age of symptom onset, adults over the age of 15 years had a higher risk (OR 2.3; 95%CI 1.6–3.3). When analysed by type of RD, a higher risk of taking more than 6 months to seek medical advice about symptoms was observed among persons affected by diseases of the nervous system (OR 1.8; 95%CI 1.3–2.4), followed by diseases of the eye and adnexa (OR 1.7, 95%CI 1.2–2.5). Conversely, shorter times were recorded for patients affected by diseases of the musculoskeletal system and connective tissue (OR 0.5, 95%CI 0.3–0.8). 

In the case of risk of diagnostic delay, the multivariate model (Table 3) showed this to be higher among persons who first sought medical advice from their primary health care provider (OR 2.5; 95%CI 1.9–3.3). When the diagnostic process began, a higher risk of experiencing a delay in diagnosis was observed for persons who had to travel to hospitals or specialists other than those usually consulted in their home province (OR 2.1; 95%CI 1.6–2.9), and for those who had to travel to a different AR (OR 1.7; 95%CI 1.1–2.5). Furthermore, persons who were diagnosed in any AR other than that in which symptom onset occurred, likewise registered a higher risk of experiencing diagnostic delay (OR 2.3; 95%CI 1.5–3.6). As was to be expected, the higher the frequency of visits to specialists, the longer the diagnostic delay, especially in cases where patients consulted specialists more than 10 times (OR 2.6; 95%CI 1.7–4.0). Similarly, time to diagnosis increased where patients underwent tests of different types (OR 1.3; 95%CI 1.2–1.5) and/or some RD-related surgical intervention, before obtaining the definitive diagnosis (OR 1.8; 95%CI 1.3–2.5). There was evidence to show that having been diagnosed through genetic testing, albeit being positive in terms of obtaining the diagnosis, increased time to diagnosis (OR 2.1; 95%CI 1.5–2.8). When it came to the type of RD, persons affected by diseases of the nervous system had a higher risk (OR 1.4; 95%CI 1.0–1.8) and those affected by diseases of the eye and adnexa had a lower risk of diagnostic delay (OR 0.7; 95%CI 0.5–0.9).

## 4. Discussion

Based on Spanish Rare Diseases Patient Registry data, this study is the first to give a detailed account of the diagnostic process experienced by patients living with RDs, and to estimate the PAR of the time that elapses between symptom onset, making a first medical visit, being referred to a specialist, and finally being attended to by the specialist. 

This study has highlighted some of the determinants of diagnostic delay, such as the type of RD, where the natural history of the individual diseases which make up each group influences the average obtained, though the low number of cases of each of these diseases would not allow for a robust and stable estimate to be obtained for any given RD. Persons affected by diseases of the nervous system display a higher risk of experiencing delays, due to the fact that many of these diseases are of great diagnostic complexity and that many of their clinical manifestations overlap, requiring more specific tests to arrive at a disease-specific diagnosis. Another determinant is making the first medical visit to primary health care to seek advice about symptoms. Furthermore, persons who experience diagnostic delay have a higher likelihood of being diagnosed in a region other than that in which they reside, and having to travel to other hospitals or specialists both within and outside their home AR. It is also more likely that they will have to visit specialists more often, that they will have to undergo more medical or even surgical tests, and that the definitive test of their diagnosis will be genetic. Our study thus corroborates the fact that persons who experience diagnostic delay undergo a more complex diagnostic process than those who are diagnosed within less than a year. A further finding is that the more recent the date of symptom onset is with respect to the date of study, the shorter the diagnostic delay, as other studies also report [5,18,19,20]. This may be related to the progressive improvement in knowledge and means, including genetic testing, for reaching a diagnosis, though recall bias may also have an influence (better recall of recent than of past events). 

The period of time that elapses between symptom onset and first medical visit may vary depending on: the severity of the symptoms, the capacity of the RD-affected person or caregiver to recognise these, and, especially, the type of RD [21,22]. Other factors highlighted as having an influence are the decade and age of symptom onset, with the time between symptom onset and first medical visit being longer in the case of diseases of appearance at adult age. When symptoms begin, most persons visit their primary health care provider, a standard step in the Spanish public health system, since specialist care is provided on referral from this first healthcare level. Nevertheless, a higher attributable risk of diagnostic delay is shown, not by time periods that depend on the patient, but rather by those that depend on the health care system. Specifically, taking more than 6 months between first medical visit and referral to a specialist may be affected by high patient-to-physician ratios or the short amount of time allowed for consultation. In addition to this, there is the elapse of more than 6 months between the order of referral to the specialist and actually attending the appointment, which is linked to the way the Spanish public health system operates, since shorter waits are reported in other countries [21]. A number of studies stress the fact that taking less time to seek medical advice is associated with a shorter time to diagnosis [21,23], and that patients who are initially referred to specialists are diagnosed more quickly [6]. 

According to our study, the fact of residing in a given AR in a decentralised health system exerts an influence via the variable which reflects the need to travel to other regions to obtain a diagnosis. During the diagnostic process, more than half of all persons had to travel to hospitals or specialists other than those usually consulted: 20.9% to another AR, and 42.6% more than four times. This finding is similar to that of the ENSERio study, which indicated that 24.6% travelled to another AR, and 41.2% had to do so more than five times [5]. In the European context, according to EurordisCare2, 25% had to travel to another region, 2% had to travel to another country, and 10% of people living with an RD were finally forced to move house after obtaining the diagnosis [4]. Other studies highlight the fact that such journeys are associated with a longer time to diagnosis [6,7,8,9], though it is true to say that this association is not always found [21,24,25]. Studies on specific RDs, such as Poland Syndrome, indicate that risk of diagnostic delay increases 3.5-fold in cases where diagnosis is received in a region other than the home region [26]. These journeys, which are associated with a longer diagnostic delay, arise particularly when the RD is complex and requires specialised referral centres, which are usually concentrated in certain ARs, such as Madrid and Catalonia, among others [27]. Travel or even changes of address involve an important investment of time and money, and also have effects of an emotional nature.

As was to be expected, persons who experienced diagnostic delay visited more physicians, with 24.2% visiting as many as 10 or more during their search for diagnosis. Other studies also quantify this for RDs as a whole, with an average of 7.3 medical visits and 14 tests before obtaining diagnosis [28]. Eurordis indicates that 24% of families in Spain visited more than five physicians, and 10% visited more than ten, though it notes differences between countries: the Polish and Danish patients surveyed needed more visits than did their French, Swiss and Dutch counterparts [29]. In the UK, around 7 out of 10 patients made more than three medical visits, and 22% made six or more such visits [30,31]. At paediatric ages, 38.0% consulted six or more different physicians, and 11.1% consulted ten or more [32]. In general, persons with more complex, acute, and numerous symptoms visit more specialists, something that tends to be associated with the great number of medical visits and taking longer to obtain the diagnosis. Although the performance of certain diagnostic tests shortens this time [6,21], in general the number of tests performed is significantly higher among persons who experience diagnostic delay. In terms of the consequences, a greater number of medical visits and tests performed entail a higher financial cost for the person affected by RD, his/her family, and the National Health System. Other possible consequences are hospitalisations and surgical interventions that are experienced by patients before being diagnosed, which prove to be more frequent among persons with diagnostic delay. The natural history of the disease means that certain actions or surgical interventions geared to correcting a specific problem or improving the patient’s quality of life may sometimes become a priority, thereby possibly making the diagnostic process even longer. Furthermore, almost one-third of persons were diagnosed through genetic testing; although this is a useful tool for obtaining diagnoses, such tests are extremely time-consuming because not everyone has already described mutations, making it advisable to shorten the time taken to obtain results. 

It is well known that healthcare systems are not always prepared to solve complex cases’ diagnosis problems as the ones some RD present. In order to face this, some solutions have been proposed: (i) using panels of genes with known effects that allow for testing if a patient has one of the known variants and can be diagnosed with a simple analysis; (ii) creating multidisciplinary units focus on RD; (iii) establishing networks of reference centres in which experts in different RD areas or groups work together to accelerate the diagnosis.

When it comes to comparing ours with other studies which analyse diagnostic delay by reference to symptom onset [33,34], it should be borne in mind that our study follows the IRDiRC indications, by establishing the date of first medical visit in order to ascertain the delay [11]. Similarly, it should be stressed that this study also furnished data on time elapsed from symptom onset. 

With respect to limitations, it is possible that the lack of representation of some RDs may affect the results shown, since RDs are addressed as a whole. Furthermore, the heterogeneity of the natural history of the RDs included might also exert an influence. Another limitation of the study is the change in case definition, clinical practice and genetic diagnosis of RD during the study period. However, this limitation is a constant within the framework of a group of diseases that are characterized by high heterogeneity even when they are not entirely new and unknown. Insofar as form is concerned, participation was affected by the restrictive measures implemented during the COVID-19 pandemic, so that the digital divide was partially reduced. Lastly, though the response rate was in line with what is to be expected for these types of studies, the fact that persons with diagnostic delay might have a greater interest in participating means that there may be a greater number of cases than controls. 

Lastly, this study’s main strength lies in its description of the diagnostic process and its determinants in Spain, being the first of its kind to use a nationwide registry open to any RD, with confirmed diagnoses in clinical reports, as its data source. Likewise, it enjoyed wide-ranging participation by RD-affected persons and their families, thanks to their being highly motivated to contribute to research. In addition, the participation of older persons and those who were visually handicapped was facilitated by using specially adapted forms along with telephone and/or in-person surveys, with the aim of mitigating possible selection biases.

## 5. Conclusions

This study is the first to address the diagnostic process of people living with RDs in Spain, based on data sourced from a national patient registry, in collaboration with FEDER and CREER. It shows that the diagnostic process undergone by people living with RDs is complex, particularly among those who experience diagnostic delay. In addition, it identified some of the determinants associated with diagnostic delay, such as making the first medical visit to the general practitioner, having to travel within the same province or to another AR, visiting more than 10 specialists, or being diagnosed in a region other than that of residence. This study also highlights the fact that the period of time posing the highest attributable risk of diagnostic delay is that of referral to the specialist, something that depends on the health system. Future in-depth studies are needed to continue the work of determining these factors in different types of RDs, along with the implications which this diagnostic process has for the lives of persons affected by RDs and their families, at a social, educational, occupational, psychological, and financial level.

## Figures and Tables

**Table 1:** Diagnostic process of people living with an RD in Spain.

		Overall %(*n*) *n* = 1216	Cases %(*n*) *n* = 699	Controls %(*n*) *n* = 517	*p* Value
Sex	Men	41.2 (501)	38.9 (272)	44.3 (229)	0.059
	Women	58.8 (715)	61.1 (427)	55.7 (288)	
Type of RD	Musculoskeletal system and connective tissue	9.3 (113)	8.7 (61)	10.1 (52)	<0.001
	Blood and blood-forming organs and certain disorders involving the immune mechanism	5.1 (62)	4.1 (29)	6.4 (33)	
	Endocrine, nutritional and metabolic diseases	8.1 (99)	8.9 (62)	7.2 (37)	
	Mental and behavioural disorders	3.5 (43)	5 (35)	1.5 (8)	
	Diseases of the nervous system	25.2 (307)	28.5 (199)	20.9 (108)	
	Diseases of the eye and adnexa	16.5 (201)	12.7 (89)	21.7 (112)	
	Diseases of the circulatory system	2.5 (31)	2.1 (15)	3.1 (16)	
	Congenital malformations, deformations and chromosomal abnormalities	22.4 (272)	24.3 (170)	19.7 (102)	
	Others	7.2 (88)	5.6 (39)	9.5 (49)	
Symptom onset and medical visits					
	<15	44.1 (535)	47.4 (331)	39.5 (204)	
Age of symptom onset (years)	15–29	18.8 (228)	17.6 (123)	20.3 (105)	0.027
	30–44	21.7 (263)	21.3 (149)	22.1 (114)	
	>45	15.5 (188)	13.6 (95)	18 (93)	
Decade of symptom onset	2010–2021	39.9 (484)	32.9 (229)	49.4 (255)	<0.001
	2000–09	26.1 (316)	30.3 (211)	20.3 (105)	
	1990–99	12.4 (150)	12.3 (86)	12.4 (64)	
	1980–89	11.1 (135)	11.5 (80)	10.7 (55)	
	Until 1979	10.6 (128)	13.1 (91)	7.2 (37)	
First medical visit due to symptoms	Neonates	4 (49)	3.4 (24)	4.8 (25)	<0.001
	Primary care/paediatrics	50.9 (618)	58.2 (406)	41 (212)	
	Specialist	27.8 (338)	22.5 (157)	35 (181)	
	Emergencies/hospital	15.6 (189)	13.9 (97)	17.8 (92)	
	Other	1.7 (21)	2 (14)	1.4 (7)	
Time from symptom onset to first medical visit	<15 days	40.3 (486)	38.9 (269)	42.2 (217)	<0.001
	15 days–1 month	15.3 (185)	13.4 (93)	17.9 (92)	
	1–6 months	20.8 (251)	19.8 (137)	22.2 (114)	
	6–12 months	7.6 (92)	8.5 (59)	6.4 (33)	
	>1 year	15.9 (192)	19.4 (134)	11.3 (58)	
Time from first medical visit to referral to specialist	<15 days	30.1 (304)	21.9 (123)	40.3 (181)	<0.001
	15 days–1 month	16.5 (167)	11.4 (64)	22.9 (103)	
	1–6 months	27.7 (280)	27.9 (157)	27.4 (123)	
	6–12 months	10.9 (110)	14.6 (82)	6.2 (28)	
	>1 year	14.8 (150)	24.2 (136)	3.1 (14)	
Referral time to specialist until appointment	<15 days	29.7 (282)	20.4 (108)	41.5 (174)	<0.001
	15 days–1 month	18.9 (179)	18.5 (98)	19.3 (81)	
	1–6 months	34.5 (327)	35.5 (188)	33.2 (139)	
	6–12 months	10.2 (97)	14.9 (79)	4.3 (18)	
	>1 year	6.7 (64)	10.8 (57)	1.7 (7)	
Travel (searching for diagnosis)					
Usual hospital as first hospital for provision of healthcare	Yes	75.1 (881)	74.9 (511)	75.4 (370)	0.867
	No	24.9 (292)	25.1 (171)	24.6 (121)	
Province of first hospital and province of diagnosis	Coincide	78.7 (918)	72.5 (492)	87.3 (426)	<0.001
	Different	21.3 (249)	27.5 (187)	12.7 (62)	
Travel to a different hospital or specialist	No	42.6 (518)	32.3 (226)	56.5 (292)	<0.001
	Yes	57.4 (698)	67.7 (473)	43.5 (225)	
Travel within the same province	No	62.6 (761)	54.9 (384)	72.9 (377)	<0.001
	Yes	37.4 (455)	45.1 (315)	27.1 (140)	
No. of journeys to same province	1	17.9 (59)	13.4 (31)	28.3 (28)	<0.001
	2–3	23.9 (79)	23.4 (54)	25.3 (25)	
	4–10	35.5 (117)	33.8 (78)	39.4 (39)	
	>10	22.7 (75)	29.4 (68)	7.1 (7)	
Travel to a different province, same AR	No	86.7 (1054)	84 (586)	90.5 (468)	<0.001
	Yes	13.3 (161)	16 (112)	9.5 (49)	
No. of journeys to another province	1	25.9 (28)	16 (12)	48.5 (16)	0.004
	2–3	31.5 (34)	34.7 (26)	24.2 (8)	
	4–10	32.4 (35)	36 (27)	24.2 (8)	
	>10	10.2 (11)	13.3 (10)	3 (1)	
Travel to another AR	No	79.1 (961)	73.4 (512)	86.8 (449)	<0.001
	Yes	20.9 (254)	26.6 (186)	13.2 (68)	
No. of journeys to another AR	1	34.6 (56)	28.3 (34)	52.4 (22)	0.007
	2–3	34.6 (56)	34.2 (41)	35.7 (15)	
	4–10	21 (34)	25 (30)	9.5 (4)	
	>10	9.9 (16)	12.5 (15)	2.4 (1)	
Travel to another country	No	98.4 (1195)	97.9 (683)	99 (512)	0.109
	Yes	1.6 (20)	2.1 (15)	1 (5)	
No. of journeys to another country	1	54.5 (6)	50 (4)	66.7 (2)	0.78
	2–3	36.4 (4)	37.5 (3)	33.3 (1)	
	4–10	9.1 (1)	12.5 (1)	0 (0)	
Change of address related with search for diagnosis	Yes	3.5 (43)	3.1 (22)	4.1 (21)	0.393
	No	96.5 (1173)	96.9 (677)	95.9 (496)	
Province of symptom onset and diagnosis	Same	76.5 (927)	70.4 (489)	84.9 (438)	<0.001
	Different	23.5 (284)	29.6 (206)	15.1 (78)	
AR of symptom onset and AR of diagnosis	Same	83.8 (1015)	78.3 (544)	91.3 (471)	<0.001
	Different	16.2 (196)	21.7 (151)	8.7 (45)	
Specialists and tests (before diagnosis)					
No. of visits to specialists	0–1 (Q1)	27.3 (320)	22.1 (151)	34.4 (169)	<0.001
	2–4 (Q2)	22.3 (262)	14.8 (101)	32.8 (161)	
	5–10 (Q3)	26.2 (307)	29.9 (204)	21 (103)	
	>10 (Q4)	24.2 (284)	33.1 (226)	11.8 (58)	
No. of tests performed	0–2 (Q1)	25.4 (298)	21 (143)	31.6 (155)	<0.001
	3–7 (Q2)	25.6 (300)	19.8 (135)	33.6 (165)	
	8–19 (Q3)	24.4 (286)	26.4 (180)	21.6 (106)	
	>19 (Q4)	24.6 (289)	32.8 (224)	13.2 (65)	
No. of different specialists	0 (Q1)	18.3 (215)	16.1 (110)	21.4 (105)	<0.001
	1–2 (Q2)	33.5 (393)	26.2 (179)	43.6 (214)	
	3–5 (Q3)	23.1 (271)	23.9 (163)	22 (108)	
	>5 (Q4)	25.1 (294)	33.7 (230)	13 (64)	
No. of different tests performed	0–1 (Q1)	26.3 (308)	22.3 (152)	31.8 (156)	<0.001
	2–3 (Q2)	20.8 (244)	17.9 (122)	24.8 (122)	
	4–8 (Q3)	28.7 (337)	28.6 (195)	28.9 (142)	
	>8 (Q4)	24.2 (284)	31.2 (213)	14.5 (71)	
RD-related hospitalisations and surgical interventions (before diagnosis)					
Hospitalisations	No	62.7 (735)	56.8 (387)	70.9 (348)	<0.001
	Yes	30.7 (360)	34.8 (237)	25.1 (123)	
	Not known if RD-related	6.6 (77)	8.4 (57)	4.1 (20)	
Surgical interventions	No	77.6 (909)	72.2 (492)	84.9 (417)	<0.001
	Yes	17.9 (210)	21.9 (149)	12.4 (61)	
	Not known if RD-related	4.5 (53)	5.9 (40)	2.6 (13)	
Diagnosis					
Time to diagnosis from first medical visit (years)	<1	42.7 (735)		100 (507)	<0.001
	1–3	21.7 (257)	37.8 (258)		
	4–9	17.2 (204)	30 (204)		
	>10	18.4 (219)	32.2 (219)		
Age of diagnosis (years)	<5	32.9 (400)	30 (210)	36.8 (190)	0.027
	15–29	17.8 (216)	17 (119)	18.8 (97)	
	30–44	25.9 (315)	27.3 (191)	24 (124)	
	>45	23.4 (285)	25.6 (179)	20.5 (106)	
Definitive or confirmatory test of diagnosis	Analytical tests	6.8 (83)	6.9 (48)	6.8 (35)	<0.001
	Biopsy	11.8 (143)	11 (77)	12.9 (66)	
	Medical criterion	10.2 (124)	10.4 (73)	9.9 (51)	
	Genetic testing	31.5 (382)	38.6 (270)	21.8 (112)	
	Ophthalmological tests	8.4 (102)	5.9 (41)	11.9 (61)	
	Neurology tests	8.2 (99)	7.9 (55)	8.6 (44)	
	Radiology tests	19.8 (240)	16.3 (114)	24.6 (126)	
	More than one test	1.5 (18)	1.6 (11)	1.4 (7)	
	DK/NO	1.8 (21)	1.4 (10)	2.1 (11)	
Travel after diagnosis					
To a different hospital or specialist	Yes	53.1 (558)	54.7 (331)	50.9 (227)	0.221
	No	46.9 (493)	45.3 (274)	49.1 (219)	
Travel within the same province	Yes	53.2 (306)	54.5 (186)	51.3 (120)	0.441
	No	46.8 (269)	45.5 (155)	48.7 (114)	
No. of journeys to same province	1	7.4 (20)	7 (12)	8 (8)	0.971
	2–3	23.5 (64)	23.3 (40)	24 (24)	
	4–10	35.7 (97)	36.6 (63)	34 (34)	
	>10	33.5 (91)	33.1 (57)	34 (34)	
Travel to different province in same AR	Yes	21.7 (125)	22.3 (76)	20.9 (49)	0.700
	No	78.3 (450)	77.7 (265)	79.1 (185)	
No. of journeys to another province	1	22.1 (21)	19.3 (11)	26.3 (10)	0.068
	2–3	30.5 (29)	35.1 (20)	23.7 (9)	
	4–10	33.7 (32)	38.6 (22)	26.3 (10)	
	>10	13.7 (13)	7 (4)	23.7 (9)	
Travel to another AR	Yes	43.1 (248)	42.5 (145)	44 (103)	0.722
	No	56.9 (327)	57.5 (196)	56 (131)	
No. of journeys to another AR	1	17.9 (32)	16.7 (18)	19.7 (14)	0.577
	2–3	36.3 (65)	33.3 (36)	40.8 (29)	
	4–10	30.2 (54)	33.3 (36)	25.4 (18)	
	>10	15.6 (28)	16.7 (18)	14.1 (10)	
Travel to another country	Yes	4.5 (26)	4.7 (16)	4.3 (10)	0.812
	No	95.5 (549)	95.3 (325)	95.7 (224)	
No. of journeys to another country	1	82.4 (14)	70 (7)	100 (7)	0.279
	2–3	11.8 (2)	20 (2)	0 (0)	
	4–10	5.9 (1)	10 (1)	0 (0)	
Change of address as a consequence of diagnosis	Yes	6.6 (72)	5.8 (36)	7.7 (36)	0.206
	No	93.4 (1015)	94.2 (585)	92.3 (430)	
RD-related hospitalisations and operations after diagnosis					
Hospitalisations	Yes	25.4 (267)	23.3 (141)	28.3 (126)	0.149
	No	72.6 (763)	74.4 (450)	70.2 (313)	
	Not known if RD-related	2 (21)	2.3 (14)	1.6 (7)	
Surgical interventions	Yes	20.2 (212)	19.3 (117)	21.3 (95)	0.392
	No	77.6 (816)	78 (472)	77.1 (344)	
	Not known if RD-related	2.2 (23)	2.6 (16)	1.6 (7)	
Specialists, tests, province of follow-up and treatment after diagnosis					
No. of specialists before vs. after diagnosis	Similar	45.1 (493)	44.4 (276)	46.1 (217)	0.049
	More	44.9 (490)	43.6 (271)	46.5 (219)	
	Fewer	10 (109)	11.9 (74)	7.4 (35)	
No. of tests before vs. after diagnosis	Similar	36 (393)	35.6 (221)	36.4 (172)	0.210
	More	36.9 (403)	35.3 (219)	39 (184)	
	Fewer	27.2 (297)	29.1 (181)	24.6 (116)	
Change of treatment after diagnosis	Yes	35.6 (389)	38 (236)	32.4 (153)	0.095
	No	28.8 (315)	28.8 (179)	28.8 (136)	
	No previous treatment	35.6 (389)	33.2 (206)	38.8 (183)	
Reason for change of treatment	Having a diagnosis	50.9 (194)	59.3 (137)	38 (57)	<0.001
	Knowing the disease course of the RD	27.3 (104)	20.3 (47)	38 (57)	
	More complete medical/social report	9.2 (35)	7.8 (18)	11.3 (17)	
	Other	12.6 (48)	12.6 (29)	12.7 (19)	

Q: quartile; RD: rare disease; AR: autonomous region; DK/NO: don’t know/no opinion.

**Table 2:** Determinants associated with taking more than 6 months to attend the first medical visit from time of RD symptom onset.

Variable	Category	OR	95%CI
Age at symptom onset	Adult (>15 years)	2.3	1.6–3.3
Decade of symptom onset	2010–2021 *		
	2000–09	2.2	1.5–3.2
	1990–99	2.6	1.7–4.1
	1980–89	2.4	1.4–4.0
	Until 1979	8.2	4.9–13.7
Type of RD	Diseases of the musculoskeletal system and connective tissue	0.5	0.3–0.8
	Diseases of the nervous system	1.8	1.3–2.4
	Diseases of the eye and adnexa	1.7	1.2–2.5

* Reference group; Variables are shown whose 95%CI do not contain the value 1. Model adjusted for age of symptom onset, decade of symptom onset, and type of RD; OR: Odds ratio; RD: rare disease; n: 1182 (2.6% missing values). Hosmer-Lemeshow test *p* = 0.292.

**Table 3:** Determinants associated with diagnostic delay for RDs in Spain.

Variable	Category	OR	95%CI
Type of RD	Diseases of the nervous system	1.4	1.0–1.8
	Diseases of the eye and adnexa	0.7	0.5–0.9
First medical visit by symptom	Primary care	2.5	1.9–3.3
Travel within the same province	Yes	2.1	1.6–2.9
Travel to another AR	Yes	1.7	1.1–2.5
AR of symptom onset and AR of diagnosis	Different	2.3	1.5–3.6
Surgical interventions	Yes	1.8	1.3–2.5
No. of visits to specialists	0–1 (Q1) *		
	2–5 (Q2)	0.8	0.6–1.1
	6–10 (Q3)	1.6	1.1–2.5
	+10 (Q4)	2.6	1.7–4.0
Ratio tests performed	Ratio	1.3	1.2–1.5
Definitive or confirmatory test of diagnosis	Genetic testing	2.1	1.5–2.8

* Reference group; Variables are shown whose 95%CI do not contain the value 1. Model adjusted for sex, type of RD, age of symptom onset, first medical visit due to symptoms, travel within same province, travel to another AR, number of journeys within same province, province of symptom onset, and province of diagnosis, AR of first hospital and AR of diagnosis, number of visits to specialists, number of different specialists, ratio of tests performed (frequency/different), hospitalisations, surgical interventions, definitive or confirmatory test of diagnosis; OR: Odds ratio; Q: quartile; RD: rare disease; AR: autonomous region; n: 1150 (5.4% missing values). Hosmer-Lemeshow test *p* = 0.702.

## Data Availability

Not applicable.

## Supplementary Materials

The following supporting information can be downloaded at: https://www.mdpi.com/article/10.3390/ijerph19116456/s1, Table S1: Table showing the complete list of included RD in the study.
