# Supplementary material for: Diagnostic Process in Rare Diseases: Determinants Associated with Diagnostic Delay
Source: Int J Environ Res Public Health. 2022 May 26;19(11):6456. doi: 10.3390/ijerph19116456 (PMC9180264; doi:10.3390/ijerph19116456)
Supplement: Supplementary file 1 [file ijerph-19-06456-s001.zip › ijerph-1729495-supplementary.pdf]

Table S1. Table showing the complete list of included RD in the study.

Rare Disease Name	ORPHA Code
15q13.3 microdeletion syndrome	199318
16p13.3 microduplication syndrome	96078
17p11.2 microduplication syndrome	1713
1p36 deletion síndrome	1606
22q11.2 deletion syndrome	567
3q29 microdeletion syndrome	65286
4p16.3 microduplication syndrome	96072
Achondroplasia	15
Achromatopsia	49382
Acrodysostosis	950
Acromegaly	963
Acute intermittent porphyria	79276
Addison disease	85138
Adenosine monophosphate deaminase deficiency	45
ADNP syndrome	404448
Adult-onset cervical dystonia	420492
Adult-onset foveomacular vitelliform dystrophy	99000
Adult-onset Still disease	829
Aicardi syndrome	50
Alagille syndrome due to a JAG1 point mutation	261619
Alkaptonuria	56
Allan-Herndon-Dudley syndrome	59
Alopecia universalis	701
Alpha-1-antitrypsin deficiency	60
Alpha-sarcoglycan-related limb-girdle muscular dystrophy R3	62
Alström syndrome	64
Alternating hemiplegia of childhood	2131
American trypanosomiasis	3386
Amyotrophic lateral sclerosis	803
Angelman syndrome	72
Anorectal malformation	96346
Antiphospholipid syndrome	80
Antisynthetase syndrome	81
Arachnoid cyst	2356
Arachnoiditis	137817
Arnold-Chiari malformation type I	268882
Arnold-Chiari malformation type I/Arnold-Chiari malformation type II	268882/1136
Arnold-Chiari malformation type II	1136
Arthrogryposis multiplex congenita	1037
ATTRV122I amyloidosis	85451
Autism spectrum disorder due to AUTS2 deficiency	352490
Autoimmune hepatitis	2137
Autoimmune pancreatitis	103919
Autoimmune polyendocrinopathy type 1	3453
Autoimmune polyendocrinopathy type 2	3143
Autosomal dominant cerebellar ataxia	99
Autosomal dominant Charcot-Marie-Tooth disease type 2K	99944
Autosomal dominant chorioretinopathy-microcephaly syndrome	1432
Autosomal dominant intellectual disability-craniofacial anomalies-cardiac defects syndrome	457193
Autosomal dominant non-syndromic intellectual disability	178469
Autosomal dominant spastic paraplegia type 3	100984
Autosomal dominant spastic paraplegia type 4	100985
Autosomal erythropoietic protoporphyria	79278
Autosomal recessive cerebellar ataxia	1172
Autosomal recessive cerebelloparenchymal disorder type 3	1170
Autosomal recessive congenital ichthyosis	281097
Autosomal recessive spastic paraplegia type 76	488594
Bardet-Biedl syndrome	110
Becker muscular dystrophy	98895
Behçet disease	117
Benign concentric annular macular dystrophy	251287
Bernard-Soulier syndrome	274
Best vitelliform macular dystrophy	1243
Bilateral generalized polymicrogyria	208447
Bilateral striopallidodentate calcinosis	1980
Bipartite talus	364198
Blackfan-Diamond anemia	124
Bohring-Opitz syndrome	97297

Borjeson-Forssman-Lehmann syndrome	127
Brittle cornea syndrome	90354
Bronchiolitis obliterans with obstructive pulmonary disease	1303
Brown-Vialetto-van Laere syndrome type II	97229
Brugada syndrome	130
Bullous pemphigoid	703
Calpain-3-related limb-girdle muscular dystrophy R1	267
Cardiofaciocutaneous syndrome	1340
Carnitine palmitoyltransferase II deficiency	157
Caroli disease	53035
Catecholaminergic polymorphic ventricular tachycardia	3286
Caudal regression sequence	3027
Central areolar choroidal dystrophy	75377
Central diabetes insipidus	178029
Cerebral autosomal dominant arteriopathy-subcortical infarcts-leukoencephalopathy	136
Charcot-Marie-Tooth disease type 1A	101081
Charcot-Marie-Tooth disease/Hereditary motor and sensory neuropathy	166
CHD3-related developmental delay-speech delay-intellectual disability-abnormalities of vision-facial dysmorphism syndrome	599082
Childhood disintegrative disorder	168782
Cholesteryl ester storage disease	75234
Choroideremia	180
Chronic inflammatory demyelinating polyneuropathy	2932
Clark-Baraitser syndrome	600731
Classic Bartter syndrome	93605
Classic galactosemia	79239
Classic glucose transporter type 1 deficiency syndrome	71277
Classic mycosis fungoides	2584
Clippers	284448
Coffin-Siris syndrome	1465
Cogan syndrome	1467
Cohen syndrome	193
Combined dystonia	98203
Combined oxidative phosphorylation defect type 23	444013
Combined pituitary hormone deficiencies, genetic forms	95494
Common variable immunodeficiency	1572
Complex regional pain syndrome type 1	99995
Complex regional pain syndrome type 2	99994

Cone rod dystrophy	1872
Congenital erythropoietic porphyria	79277
Congenital factor VII deficiency	327
Congenital factor XI deficiency	329
Congenital fiber-type disproportion myopathy	2020
Congenital fibrosis of extraocular muscles	45358
Congenital glaucoma	98976
Congenital hypothyroidism	442
Congenital limbs-face contractures-hypotonia-developmental delay syndrome	562528
Congenital pulmonary lymphangiectasia	2414
Constitutional megaloblastic anemia due to vitamin B12 metabolism disorder	98396
Cornelia de Lange syndrome	199
Cowden syndrome	201
CREST syndrome	90290
Crisponi syndrome	1545
Cushing disease	96253
Cutaneous mastocytosis	66646
Cystic fibrosis	586
Dent disease	1652
Dermatomyositis	221
Desmoid tumor	873
Diffuse cutaneous mastocytosis	79456
Diffuse neonatal hemangiomatosis	2123
Disorder of fructose metabolism	308463
Disorder of urea cycle metabolism and ammonia detoxification	79167
Distal monosomy 12p	280325
Distal myotilinopathy	98911
Distal renal tubular acidosis	18
Distal spinal muscular atrophy	53739
Dravet síndrome	33069
Duane retraction syndrome	233
Duchenne muscular dystrophy	98896
Dural sinus malformation	97339
Dystrophic epidermolysis bullosa	303
Early infantile epileptic encephalopathy	1934
Early-onset generalized limb-onset dystonia	256
Early-onset non-syndromic cataract	91492
Ectrodactyly with and without other manifestations	498477
Ehlers-Danlos syndrome	98249
Eosinophilic esophagitis	73247

Eosinophilic fasciitis	3165
Eosinophilic gastroenteritis	2070
Eosinophilic granulomatosis with polyangiitis	183
Epidermolysis bullosa simplex	304
Epithelioid hemangioendothelioma	157791
Esophageal atresia	1199
Essential iris atrophy	98981
Essential thrombocythemia	3318
F12-related hereditary angioedema with normal C1Inh	100054
Fabry disease	324
Facioscapulohumeral dystrophy	269
Familial drusen	75376
Familial exudative vitreoretinopathy	891
Familial Mediterranean fever	342
Familial paroxysmal ataxia	97
Febrile infection-related epilepsy syndrome	163703
Female restricted epilepsy with intellectual disability	101039
Fibrodysplasia ossificans progressiva	337
Focal, segmental or multifocal dystonia	1866
FOXG1 syndrome	561854
Fragile X syndrome	908
Friedreich ataxia	95
Gastrointestinal stromal tumor	44890
Gaucher disease type 1	77259
Gitelman syndrome	358
Global developmental delay-neuro-ophthalmological abnormalities-seizures-intellectual disability syndrome	488613
Glutaryl-CoA dehydrogenase deficiency	25
Glycogen storage disease due to muscle glycogen phosphorylase deficiency	368
GNAO1-related developmental delay-seizures-movement disorder spectrum	592564
Goldenhar syndrome	374
Gorlin syndrome	377
Granulomatosis with polyangiitis	900
GRIN2B-related developmental delay, intellectual disability and autism spectrum disorder	589547
Gyrate atrophy of choroid and retina	414
Hemolytic anemia due to red cell pyruvate kinase deficiency	766
Hereditary angioedema type 1	100050
Hereditary angioedema type 2	100051
Hereditary fructose intolerance	469
Hereditary hemorrhagic telangiectasia	774
Hereditary motor and sensory neuropathy type 5	64751
Hereditary neuropathy with liability to pressure palsies	640
Hereditary spastic paraplegia	320335
Hidrotic ectodermal dysplasia	189
Hinman syndrome	84085
Hirschsprung disease	388
Holt-Oram syndrome	392
Horizontal gaze palsy with progressive scoliosis	2744
Huntington disease	399
Hyperlipoproteinemia type 1	411
Hypermobile Ehlers-Danlos syndrome	285
Hypohidrotic ectodermal dysplasia	238468
Hypoxanthine guanine phosphoribosyltransferase partial deficiency	79233
Ichthyosis	79354
Idiopathic achalasia	930
Idiopathic hypersomnia	33208
Idiopathic intracranial hypertension	238624
Idiopathic multicentric Castleman disease	570431
Idiopathic pleuroparenchymal fibroelastosis	494428
Idiopathic pulmonary arterial hypertension	275766
Idiopathic pulmonary fibrosis	2032
IgG4-related retroperitoneal fibrosis	49041
Immune thrombocytopenia	3002
Inclusion body myopathy with Paget disease of bone and frontotemporal dementia	52430
Indolent systemic mastocytosis	98848
Intellectual disability syndrome due to a DYRK1A point mutation	464311
Intellectual disability-cataracts-calcified pinnae-myopathy syndrome	3042
Intellectual disability-craniofacial dysmorphism-cryptorchidism syndrome	329224
Intellectual disability-expressive aphasia-facial dysmorphism syndrome	436151
Intellectual disability-facial dysmorphism syndrome due to SETD5 haploinsufficiency	404440
Intellectual disability-macrocephaly-hypotonia-behavioral abnormalities syndrome	457279
Interstitial cistitis	37202
Inverted duplicated chromosome 15 syndrome	3306

IRF2BPL-related regressive neurodevelopmental disorder-dystonia-seizures syndrome	597623
Isolated aniridia	250923
Isolated biliary atresia	30391
Isolated complex I deficiency	2609
Isolated complex III deficiency	1460
Isolated congenital hypogonadotropic hypogonadism	238666
Isolated corpus callosum agenesis	200
Isolated cytochrome C oxidase deficiency	254905
Isolated Klippel-Feil syndrome	2345
Isolated optic neuritis	499096
Isolated scaphocephaly	35093
Juvenile dermatomyositis	93672
Juvenile overlap myositis	329894
Juvenile xanthogranuloma	158000
Kabuki syndrome	2322
KBG syndrome	2332
KCNQ2-related epileptic encephalopathy	439218
Kennedy disease	481
Kleine-Levin syndrome	33543
Klippel-Trénaunay syndrome	90308
Koolen-De Vries syndrome	96169
Lamb-Shaffer syndrome	530983
Laminin subunit alpha 2-related congenital muscular dystrophy	258
Langerhans cell histiocytosis	389
Larsen syndrome	503
Leber congenital amaurosis	65
Leber hereditary optic neuropathy	104
Legg-Calvé-Perthes disease	2380
Leigh syndrome	506
Limb-girdle muscular dystrophy	263
Localized dystrophic epidermolysis bullosa, acral form	158673
Localized scleroderma	90289
Lymphangioleiomyomatosis	538
Lynch syndrome	144
Marfan syndrome	558
McLeod neuroacanthocytosis syndrome	59306
Medium chain acyl-CoA dehydrogenase deficiency	42
Megalencephalic leukoencephalopathy with subcortical cysts	2478
Megalencephaly	2477
Megalencephaly-capillary malformation-polymicrogyria syndrome	60040
Melkersson-Rosenthal syndrome	2483
Melorheostosis	2485
Menke-Hennekam syndrome	592574
Mesial temporal lobe epilepsy with hippocampal sclerosis	99701
Metaphyseal chondrodysplasia, Jansen type	33067
Methylmalonic acidemia with homocystinuria, type cblC	79282
Microcornea-posterior megalolenticonus-persistent fetal vasculature-coloboma syndrome	231736
Mitochondrial myopathy	206966
Mitochondrial myopathy-lactic acidosis-deafness syndrome	2597
Mixed connective tissue disease	809
Monoclonal mast cell activation syndrome	529468
Monomelic amyotrophy	65684
Monosomy 18p	1598
Monosomy 22q13.3	48652
Monosomy 5p	281
Monosomy 9p	261112
Motor neuron disease	98503
Mucopolysaccharidosis type 2	580
Mucopolysaccharidosis type 3	581
Multiminicore myopathy	598
Multiple endocrine neoplasia type 2ª	247698
Multiple endocrine neoplasia type 2B	247709
Multiple myeloma	29073
Multiple osteochondromas	321
Muscular dystrophy	98473
Myasthenia gravis	589
Myelomeningocele	93969
Myhre syndrome	2588
Myoclonus-dystonia syndrome	36899
Myopic macular degeneration	178493
Narcolepsy type 1	2073
Narcolepsy type 1	2073
Narcolepsy type 2	83465
Neuralgic amyotrophy	2901
Neuroacanthocytosis	263440
Neurofibromatosis type 1	636
Neurofibromatosis type 2	637
Neuromyelitis optica spectrum disorder	71211
Nicolaides-Baraitser syndrome	3051

Niemann-Pick disease type B	77293
Non-acquired panhypopituitarism	90695
Non-specific early-onset epileptic encephalopathy	442835
Noonan syndrome	648
Noonan syndrome with multiple lentigines	500
Noonan syndrome-like disorder with loose anagen hair	2701
NRXN1-related severe neurodevelopmental disorder-motor stereotypies-chronic constipation-sleep-wake cycle disturbance	600663
Oculopharyngeal muscular dystrophy	270
Oligodontia	99798
Ollier disease	296
Opsoclonus-myoclonus syndrome	1183
Oromandibular dystonia	93958
Osteochondritis dissecans	2764
Osteogenesis imperfecta	666
Osteogenesis imperfecta type 4	216820
Overlapping connective tissue disease	251312
Paroxysmal nocturnal hemoglobinuria	447
Partial deletion of the long arm of chromosome 18	262146
Partial deletion of the long arm of chromosome 2	262010
Partial trisomy/tetrasomy of the short arm of chromosome 12	262658
Pelizaeus-Merzbacher disease	702
Pemphigus vulgaris	704
Perineural cyst	65250
Peroxisome biogenesis disorder	79189
Peutz-Jeghers syndrome	2869
Phenylketonuria	716
Pigmented villonodular synovitis	66627
Pitt-Hopkins syndrome	2896
PMM2-CDG	79318
Poland syndrome	2911
Polyarteritis nodosa	767
Polycythemia vera	729
Porphyria variegata	79473
Postpoliomyelitis syndrome	2942
Postural orthostatic tachycardia syndrome due to NET deficiency	443236
Potocki-Shaffer syndrome	52022
Prader-Willi syndrome	739
Primary ciliary dyskinesia, Kartagener type	98861

Primary erythromelalgia	90026
Primary hypereosinophilic syndrome	314950
Primary progressive aphasia	95432
Progressive cone dystrophy	1871
Progressive hemifacial atrophy	1214
Prolidase deficiency	742
Proximal 16p11.2 microdeletion syndrome	261197
Pseudoachondroplasia	750
Pseudohypoparathyroidism	97593
Pseudohypoparathyroidism type 1A	79443
Pseudoxanthoma elasticum	758
Pudendal neuralgia	60039
Pulmonary alveolar microlithiasis	60025
Pulmonary arterial hypertension associated with another disease	275791
Punctate palmoplantar keratoderma	307967
Radial hemimelia	93321
Rapid-onset dystonia-parkinsonism	71517
Rare dystonia	68363
Rare genetic dystonia	391799
Rare hereditary ataxia	183518
Rare inflammatory bowel disease	104012
Rare pulmonary hypertension	71198
Recessive X-linked ichthyosis	461
Renal nutcracker syndrome	71273
Reticular dysgenesis	335
Retinitis pigmentosa	791
Retinoblastoma	790
Rett syndrome	778
Rheumatoid factor-negative polyarticular juvenile idiopathic arthritis	85408
Rubinstein-Taybi syndrome	783
SAPHO syndrome	793
Sarcoidosis	797
Selective IgM deficiency	331235
Semicircular canal dehiscence syndrome	420402
Serpiginous choroiditis	35686
Severe intellectual disability and progressive spastic paraplegia	280763
Severe intellectual disability-poor language-strabismus-grimacing face-long fingers syndrome	363686
Severe intellectual disability-progressive spastic diplegia syndrome	404473
Shwachman-Diamond syndrome	811
Sjögren-Larsson syndrome	816

Smith-Magenis syndrome	819
Sotos syndrome	821
Spinocerebellar ataxia type 3	98757
Spinocerebellar ataxia type 38	423296
Sporadic adult-onset ataxia of unknown etiology	247234
Sprengel deformity	3181
Stargardt disease	827
Steinert myotonic dystrophy	273
Steroid-responsive encephalopathy associated with autoimmune thyroiditis	83601
Stevens-Johnson syndrome	36426
Stiff person spectrum disorder	3198
Stromal corneal dystrophy	98626
Stromal corneal dystrophy	98676
Sturge-Weber syndrome	3205
STXBP1-related encephalopathy	599373
Succinic semialdehyde dehydrogenase deficiency	22
Sympathetic ophthalmia	79098
Syndrome with corpus callosum agenesis/dysgenesis as a major feature	199639
Syndromic genetic keratoconus/Syndromic keratoconus	522564/ 98623
SYNGAP1-related developmental and epileptic encephalopathy	544254
Syringomyelia	3280
Systemic capillary leak syndrome	188
Systemic lupus erythematosus	536
Systemic mastocytosis	2467
Systemic sclerosis	90291
Systemic-onset juvenile idiopathic arthritis	85414
Tetrasomy 9p	3310
Thalidomide embryopathy	3312
Thomsen and Becker disease	614
Thoracic outlet syndrome	97330
Toxic oil syndrome	227972
Treacher-Collins syndrome	861
Trigeminal neuralgia	221091
Trisomy 18	3380
Trisomy X	3375
Tuberous sclerosis complex	805
Tumor necrosis factor receptor 1 associated periodic syndrome	32960
Turner syndrome	881
Unclassified autoinflammatory síndrome	324936
Usher syndrome	886
Usher syndrome type 1	231169
Usher syndrome type 2	231178
Uveal melanoma	39044
VACTERL/VATER association	887
Vascular Ehlers-Danlos syndrome	286
Vestibular schwannoma	252175
Von Willebrand disease type 1	166078
WAGR syndrome	893
Walker-Warburg syndrome	899
White matter hypoplasia-corpus callosum agenesis-intellectual disability syndrome	3207
Williams syndrome	904
Wilson disease	905
Wolf-Hirschhorn syndrome	280
X-linked adrenoleukodystrophy	43
X-linked Charcot-Marie-Tooth disease	64747
X-linked creatine transporter deficiency	52503
X-linked non-syndromic intellectual disability	777
Xeroderma pigmentosum	910
